# Hydrophilic and Underwater Oleophobic Chitosan/Polyvinyl Alcohol/Cellulose Aerogel for Efficient Oil/Water Emulsion Separation

**DOI:** 10.3390/gels12060531

**Published:** 2026-06-12

**Authors:** Daning Lang, Mengyuan Yan, Ming Shi, Shixue He, Ronglan Wu

**Affiliations:** 1 Key Laboratory of Oil & Gas Fine Chemicals, Ministry of Education & Xinjiang Uygur Autonomous Region, School of Chemical Engineering, Xinjiang University, Urumqi 830017, China; 2 School of Mechanical Engineering & Center for Post-Doctoral Studies of Mechanical Engineering, Xinjiang University, Urumqi 830017, China

**Keywords:** cellulose aerogel, chitosan, polyvinyl alcohol, underwater oleophobicity, oil/water separation, emulsion separation

## Abstract

Oily wastewater, especially stable oil-in-water (O/W) emulsions, threatens aquatic ecosystems and is difficult to treat using conventional separation technologies. Herein, a hydrophilic and underwater oleophobic chitosan/polyvinyl alcohol (PVA)/cellulose aerogel (CPCG) was fabricated through a facile one-pot dip-coating strategy. Cellulose aerogel (CG) was prepared by low-temperature dissolution, network reinforcement, washing, and freeze-drying, before being coated with a cross-linked CS/PVA layer using glutaraldehyde, followed by NaOH solidification. SEM revealed a honeycomb-like cellulose framework uniformly covered by the CS/PVA coating, which improved the structural integrity of the skeleton. FT-IR and TG analyses supported the successful construction of the coating and the enhanced thermal stability of CPCG. CPCG displayed a high underwater oil contact angle of 153.8°, which remained above 153° after 30 min, indicating robust underwater oil repellency. Wet CPCG retained 99% of its original height after 30 compression–recovery cycles. Owing to the stable hydration layer, interconnected channels, and improved wet-state resilience, CPCG efficiently separated light and heavy oil/water mixtures and various O/W emulsions. The separation efficiencies for different emulsions were above 99%, and CPCG retained about 93% efficiency after ten cyclohexane/water emulsion separation cycles. This work provides a green and scalable route for constructing biomass-based aerogels for oily wastewater treatment.

## 1. Introduction

Natural polymer aerogels have attracted increasing attention as sustainable materials for water treatment because of their renewable origin, low density, high porosity, tunable surface chemistry, and interconnected three-dimensional networks [1,2]. Among them, cellulose-based aerogels are particularly attractive because cellulose is abundant, biodegradable, mechanically tailorable, and rich in hydroxyl groups, which provide active sites for surface modification and wettability regulation [3,4]. In oily wastewater treatment, aerogels can be designed either as hydrophobic/oleophilic absorbents for floating oil and organic solvent recovery or as hydrophilic and underwater oleophobic filters for O/W emulsion separation, depending on their surface chemistry and pore architecture [5].

However, pristine natural polymer aerogels often suffer from insufficient structural stability, pore-wall deformation and unstable wettability during long-term oil/water separation [6]. Surface modification has therefore been widely used to regulate interfacial wettability. Low-surface-energy components, including siloxanes, long-chain alkyl groups, fluorinated compounds, and hydrophobic nanoparticles, have been introduced into cellulose-, CS-, and other biomass-derived aerogels to construct superhydrophobic/superoleophilic surfaces [7,8]. For example, all-biomass aerogels and cellulose/CS-based superhydrophobic aerogels have recently shown excellent oil adsorption capacity, recyclability, and mechanical elasticity [9]. Nevertheless, these hydrophobic modification strategies are mainly suitable for oil adsorption and recovery, whereas the separation of stable O/W emulsions remains more challenging because emulsified oil droplets can adhere to pore walls, block transport channels, and reduce separation durability. In addition, non-uniform coating distribution and weak interfacial adhesion may further compromise the long-term reliability of modified aerogels under repeated filtration or compression [10].

Unlike oil absorption, hydration-mediated separation based on hydrophilic and underwater oleophobic surfaces provides an effective route for gravity-driven O/W emulsion separation [11]. After pre-wetting, hydrophilic groups within the skeleton bind water molecules and form a hydration layer on the pore walls. This water-rich interfacial layer allows the aqueous phase to pass through while preventing oil droplets from contacting the solid surface underwater [12]. Recent studies have demonstrated that sugarcane-based superhydrophilic membranes, cellulose nanofiber aerogels, and cellulose-based composite aerogels can achieve efficient O/W emulsion separation by combining hydrophilic surface chemistry with hierarchical porous structures [13]. In addition, asymmetric PVA/cellulose aerogels and biodegradable CS/PVA nanofiber membranes further confirm that hydrophilic polymer networks are beneficial for improving emulsion separation efficiency, antifouling ability, and cyclic stability [14,15]. Despite these achievements, many reported hydrophilic membranes are essentially two-dimensional separation layers and therefore lack the three-dimensional resilience, wet-state shape recovery, and structural adaptability required for repeated filtration [16].

CS, a cationic natural polysaccharide obtained by the deacetylation of chitin, contains abundant amino and hydroxyl groups and can provide strong hydration capability and interfacial interactions for constructing underwater oil-repellent surfaces [17]. Cellulose/CS aerogels and chitin nanofibril/CS aerogels have shown potential in oil/water separation and elasticity regulation [18]. However, CS-rich materials may swell in water and suffer from insufficient mechanical robustness during long-term filtration or repeated compression, which limits their practical use [19].

PVA, with abundant hydroxyl groups, good film-forming ability, flexibility, and compatibility with polysaccharides, can improve the continuity and mechanical stability of CS-based coatings [20,21]. In addition, glutaraldehyde (GA) can crosslink CS and PVA by reacting with amino and hydroxyl groups, thereby forming a more stable polymer network and suppressing coating swelling in aqueous environments [15]. Therefore, constructing a cross-linked CS/PVA coating on cellulose aerogels is expected to combine the hydration capability of CS, the film-forming and toughening effects of PVA, and the interconnected porous support of cellulose aerogels.

Despite these advances, it remains difficult to construct a continuous and stable hydrophilic coating on both the external surface and internal pore walls of cellulose aerogels without significantly blocking the interconnected pores. Such a structure is critical for achieving high water flux, low fouling, wet-state resilience, and recyclable O/W emulsion separation [22,23,24]. Therefore, a simple, green, and scalable strategy is still needed to fabricate biomass-based aerogels that combine stable hydration interfaces, three-dimensional mechanical recoverability, and durable separation performance.

In this work, a CPCG was developed using CG as a lightweight porous skeleton and CS/PVA as a hydrophilic coating system. Through a simple dip-coating, GA crosslinking, and NaOH-assisted solidification process, the CS/PVA coating was introduced onto both the external surface and internal pore walls of the cellulose framework. This design aims to enhance pore-wall hydration, coating stability, and wet-state resilience while preserving interconnected channels for water transport. The resulting CPCG was systematically evaluated in terms of morphology, chemical structure, thermal stability, underwater oleophobicity, wet-state compression resilience, oil/water mixture separation, O/W emulsion separation, and cyclic durability. Furthermore, its underwater oil contact angle, wet-state compression recovery, residual oil concentration, emulsion flux, and cyclic filtration performance were systematically analyzed, and the overall separation performance of CPCG was compared with that of related aerogel-based separation materials to demonstrate its potential for efficient, low-energy, and reusable emulsion separation.

## 2. Results and Discussion

### 2.1. Morphology Analysis

**Figure 1:**
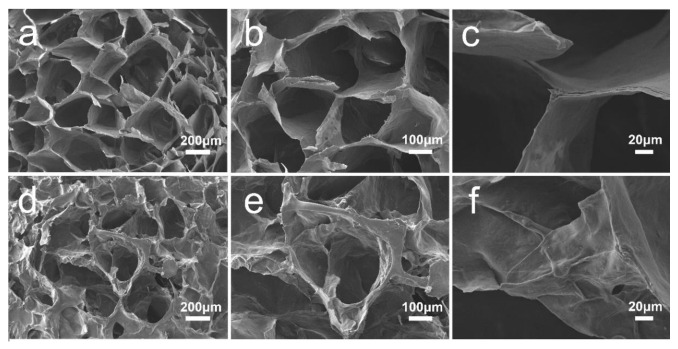
SEM images of (**a**–**c**) CG and (**d**–**f**) CPCG.

The microstructures of CG and CPCG were first examined by SEM to clarify the effect of the CS/PVA coating on the porous cellulose framework. As shown in Figure 1a–c, CG displayed a typical honeycomb-like porous architecture produced by ice-templated freeze-drying. Such interconnected pores are commonly formed by ice-crystal-templated growth and subsequent sublimation, and they provide continuous transport pathways for water permeation during separation [25]. Nevertheless, obvious fractured regions were observed in the cellulose skeleton, indicating that pristine CG possessed a relatively brittle framework. This brittleness can be attributed to the rigid cellulose network generated during solvent exchange and freeze-drying, where capillary stress and ice-crystal growth may induce local shrinkage, pore-wall rupture, and structural defects. After modification with the CS/PVA/GA coating, the surface morphology changed noticeably (Figure 1d–f). A continuous polymer layer was formed on both the external surface and internal pore walls of the cellulose framework. The coating partially covered the rough cellulose skeleton and bridged fractured regions, thereby improving framework continuity without completely blocking the interconnected pores. This morphology is important because excessive coating would reduce water permeability, whereas insufficient coating would fail to stabilize the hydrated interface. The improved structural integrity of CPCG can be attributed to multiple interactions among cellulose, CS, and PVA. The hydroxyl groups of cellulose and PVA, together with the amino and hydroxyl groups of CS, can generate abundant hydrogen bonds, while GA-induced interactions, mainly Schiff base formation with CS and possible interactions with hydroxyl-containing components, further strengthen the CS/PVA layer. Therefore, the coating acts not only as a hydrophilic interface modifier but also as a flexible reinforcing layer that protects the brittle cellulose skeleton. This coupled structural and interfacial regulation provides a basis for the enhanced wet-state resilience and underwater oleophobicity of CPCG.

### 2.2. Chemical Structure

**Figure 2:**
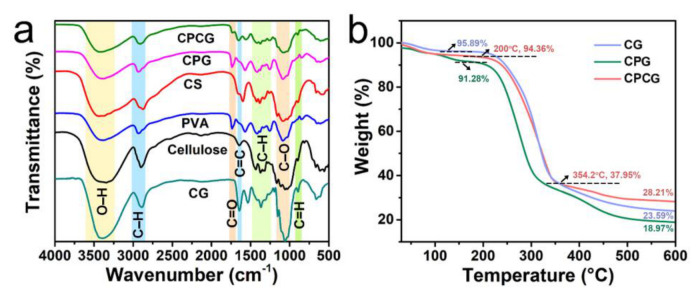
(**a**) FT−IR spectra and (**b**) TG curves of the aerogel.

FT-IR spectroscopy was used to examine the chemical composition and interfacial interactions of the aerogels (Figure 2a). All samples exhibited a broad absorption band at 3200–3700 cm^−1^, corresponding to O–H stretching vibrations from cellulose, CS, and PVA. The bands at 3000–2850 cm^−1^ were assigned to C-H stretching vibrations, while the characteristic peaks in the range of 1260–1000 cm^−1^ were related to C–O and C–O–C stretching vibrations [26]. Compared with CG, CPCG showed obvious changes in the O–H and C-O-related regions [27], indicating enhanced intermolecular interactions among cellulose, CS, and PVA. The band near 1650 cm^−1^ may arise from the overlapping contributions of absorbed water bending [28], amide-related groups in CS, and possible GA-induced interactions. Moreover, the broadened O–H absorption band suggests enhanced hydrogen bonding among cellulose, CS, and PVA. The above results confirm the CS/PVA coating and the presence of abundant hydrophilic functional groups.

### 2.3. Thermal Stability

The thermal stability of CPCG was further evaluated by TG analysis, as shown in Figure 2b. Slight weight loss below 200 °C was observed, with a residual mass of 94.36%, which can be mainly attributed to the evaporation of physically adsorbed water. The major weight loss occurred at approximately 200–350 °C, with the residual mass decreasing to 37.95%. This stage is associated with the thermal decomposition of polymer chains, including dehydration, glycosidic bond cleavage and backbone degradation [28]. In comparison, CPG exhibited a lower decomposition temperature range of approximately 200–300 °C and a lower residual mass of 18.97%, indicating the relatively poor thermal stability of the pure CS/PVA coating network. By contrast, the cellulose framework in CG and CPCG contributed to improved thermal stability. Notably, CPCG retained a relatively high residual mass of 28.21% at high temperature, suggesting that the cross-linked CS/PVA coating promoted char formation and enhanced the thermal robustness of the composite aerogel. These results demonstrate that the introduction of the CS/PVA coating not only regulates the surface chemical properties of the aerogel but also contributes to the improved thermal stability of CPCG.

### 2.4. Wettability

**Figure 3:**
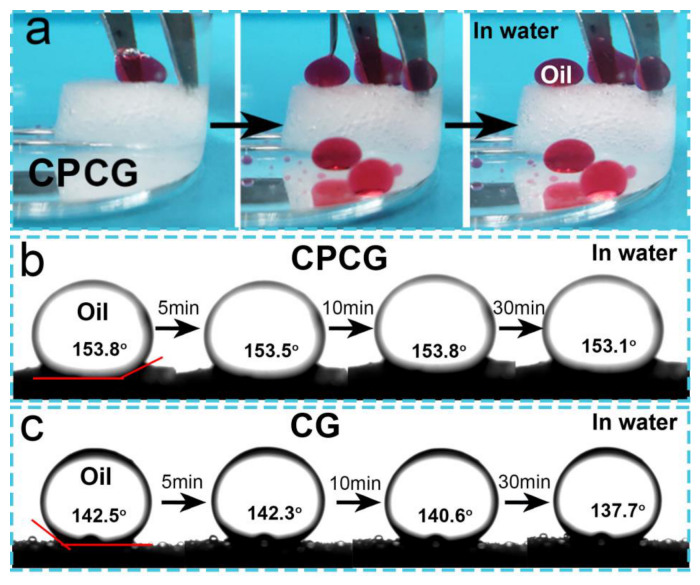
(**a**) Underwater oleophobic behavior of CPCG; underwater oil (chloroform) contact angles of (**b**) CPCG and (**c**) CG.

**Figure 4:**
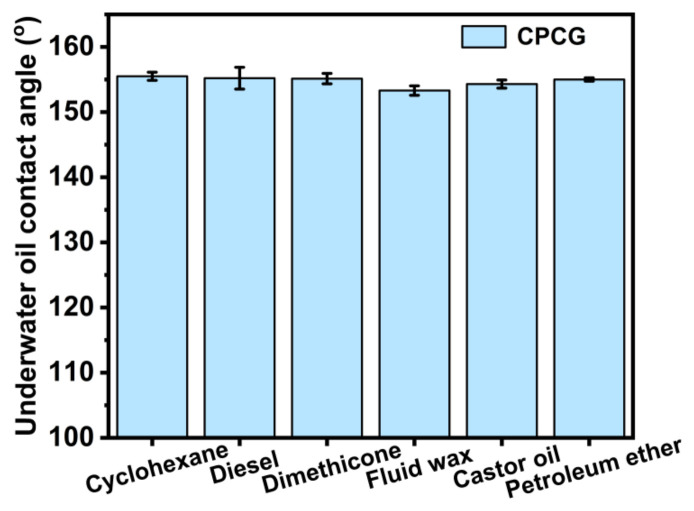
Underwater oil contact angles of CPCG.

Using chloroform as a representative oil phase, the underwater oil repellency of the aerogels was evaluated, as shown in Figure 3. In water, the chloroform droplet maintained a nearly spherical shape on the CPCG surface without obvious spreading or wetting, giving a high underwater oil contact angle of 153.8° (Figure 3a). After contact for 30 min, the contact angle changed only slightly, confirming the stable underwater oil-repellent property of CPCG (Figure 3b). In contrast, CG showed relatively poor stability. Its initial underwater oil contact angle was 142.5°, which gradually decreased to 137.7° after 30 min, indicating weaker resistance to oil adhesion and wetting (Figure 3c). The enhanced underwater oleophobicity of CPCG can be attributed to the CS/PVA composite coating. The coating increases the surface roughness and introduces abundant hydrophilic groups, such as amino and hydroxyl groups. In an aqueous environment, these groups rapidly adsorb water molecules and form a stable hydrated layer on the aerogel surface and pore walls. This hydrated layer acts as an effective barrier to prevent direct oil–solid contact, thereby suppressing oil adhesion, spreading, and penetration into the porous framework. Furthermore, CPCG maintained underwater oil contact angles above 152° toward six different oils and organic solvents (Figure 4), demonstrating its excellent universal underwater superoleophobicity and potential for oil/water separation under complex conditions.

### 2.5. Mechanical Compression Resilience

**Figure 5:**
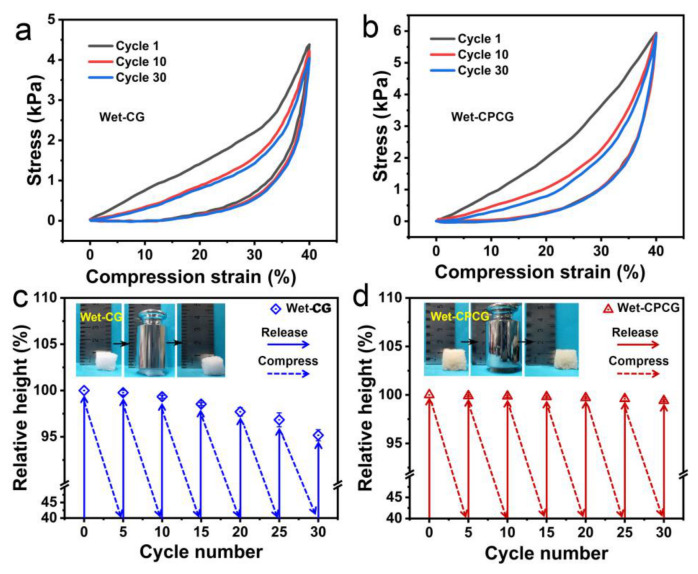
Compressive stress–strain curves of (**a**) CG and (**b**) CPCG; compression resilience of (**c**) CG and (**d**) CPCG.

Figure 5 shows the cyclic compression behavior of wet CG and CPCG aerogels under 40% strain. Both aerogels exhibited typical compression behavior of porous materials (Figure 5a,b). At low strain, the deformation was mainly associated with elastic bending of the pore walls, resulting in a gradual increase in stress. With increasing strain, progressive pore compaction and enhanced pore-wall contact led to a rapid increase in stress. For wet CG, the peak stress in the first compression cycle was 4.3 kPa and decreased continuously after 10 and 30 cycles, indicating gradual structural weakening under repeated wet-state compression (Figure 5a). In contrast, CPCG exhibited a higher initial peak stress of 6.0 kPa under the same strain, suggesting enhanced framework strength after CS/PVA modification (Figure 5b). Moreover, after 30 compression cycles, CPCG showed only slight stress attenuation, and the loading–unloading curves largely overlapped, indicating excellent mechanical stability and energy dissipation capability in the wet state.

The height recovery results further confirmed the superior cyclic resilience of CPCG. After 30 compression–recovery cycles, CG retained only 95% of its original height (Figure 5c), whereas CPCG maintained nearly 99% of its initial height (Figure 5d). This result demonstrates that CPCG possesses better wet-state fatigue resistance and shape recovery ability than CG. This can be attributed to the reinforcing and stress-dissipating effects of the cross-linked CS/PVA coating. In water, the native hydrogen-bonding network of cellulose is easily weakened by water molecules, leading to local pore-wall collapse and irreversible deformation during repeated compression. After modification, the CS/PVA coating uniformly covers the cellulose pore walls and acts as a flexible reinforcing layer that bridges the porous skeleton, disperses external stress, and reduces local stress concentration. Meanwhile, the hydrogen-bonding interactions among cellulose, chitosan, and PVA, together with CA-induced interactions, help stabilize the three-dimensional porous framework during cyclic deformation. The excellent wet-state resilience of CPCG is beneficial for maintaining stable oil/water separation performance. A well-preserved porous structure ensures continuous water transport channels, while the intact hydrophilic coating helps maintain the hydrated layer and underwater superoleophobicity during repeated filtration. Therefore, compared with unmodified CG, CPCG exhibits superior structural durability and cyclic separation stability under wet operating conditions.

### 2.6. Oil/Water and Emulsion Separation Performance

**Figure 6:**
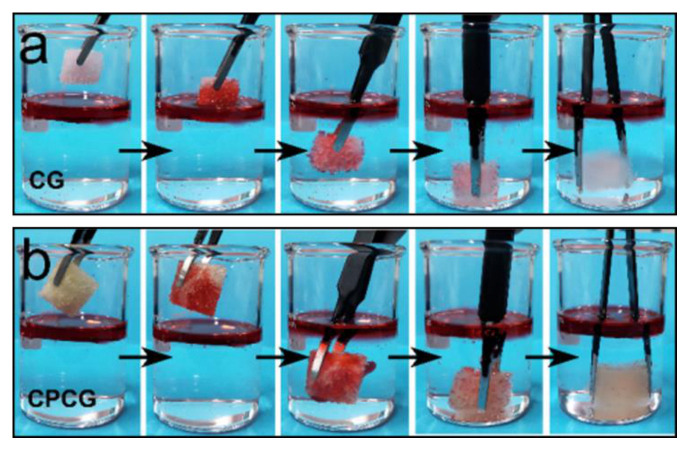
Oil adsorption and desorption processes of (**a**) CG and (**b**) CPCG.

As shown in Figure 6a, dry CG rapidly absorbed Sudan III-stained n-hexane upon contact with the oil layer, which was mainly driven by capillary force within the porous network. When the oil-loaded aerogel was immersed in water and gently squeezed, the absorbed n-hexane was released, and the aerogel gradually recovered its original shape. CPCG exhibited a similar oil absorption-release behavior under dry conditions (Figure 6b). These results indicate that the open porous structure enables the aerogels to absorb oil in the dry state. However, after being wetted with water, the hydrophilic framework is preferentially occupied by water, forming a hydrated barrier on the pore walls and surface. This wetting transition changes the aerogel from an oil-absorbing porous material into an underwater oil-repellent separation layer, allowing it to exhibit distinct functions under dry and wet conditions.

**Figure 7:**
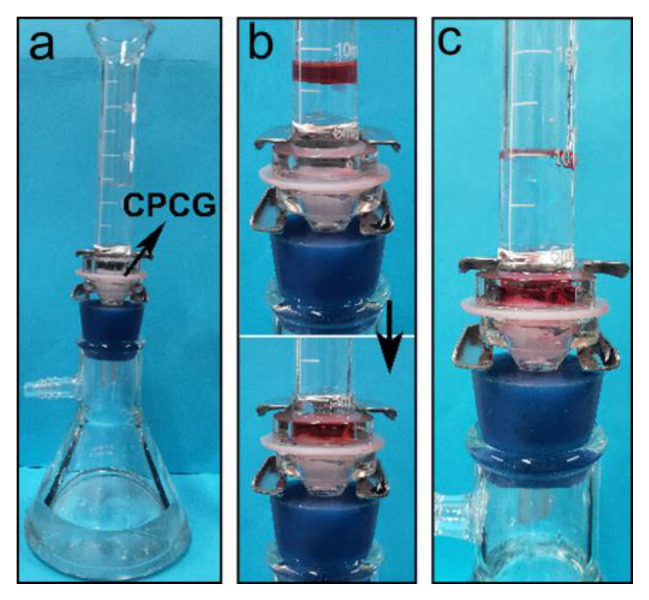
(**a**) Self-made oil/water separation device; (**b**) separation of water and floating oil by CPCG; (**c**) interception of underwater heavy oil by CPCG.

The oil/water mixture separation behavior was further evaluated using n-hexane/water as a representative light-oil system. As shown in Figure 7a,b, when the mixture was poured into the separation device, water rapidly permeated through the pre-wetted aerogel membrane and was collected in the receiving bottle, whereas the upper n-hexane phase was effectively blocked on the aerogel surface. The whole separation process was driven solely by gravity without external pressure, suggesting the potential of the aerogel membrane for low-energy oil/water separation.

To verify its density-independent separation capability, chloroform was selected as a representative heavy oil. As shown in Figure 7c, the pre-wetted aerogel membrane also effectively blocked the underlying chloroform phase at the underwater oil-repellent interface. The successful interception of both floating n-hexane and sinking chloroform demonstrates that the separation behavior is governed primarily by interfacial wettability rather than oil density. The water-rich hydrated layer formed on the hydrophilic framework serves as an effective oil-blocking barrier, enabling CPCG to separate oil/water mixtures containing oils with different densities.

**Figure 8:**
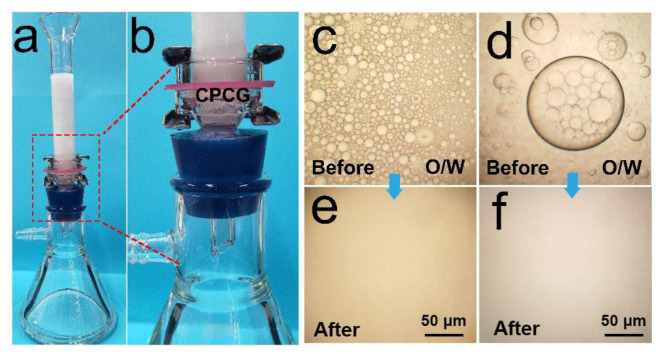
(**a**,**b**) Emulsion separation device; optical microscopy images of (**c**,**e**) cyclohexane/water emulsions and (**d**,**f**) castor oil/water emulsions.

**Figure 9:**
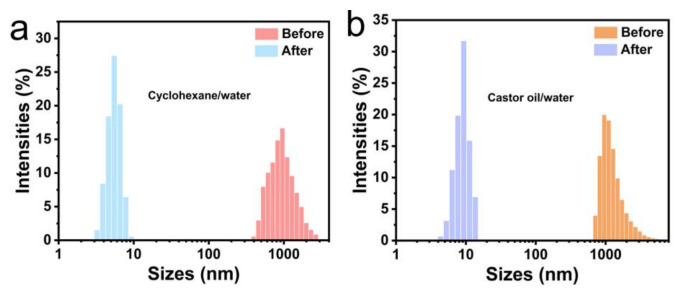
Particle size distribution before and after emulsion separation: (**a**) cyclohexane/water emulsion; (**b**) castor oil/water emulsion.

The emulsion separation performance of CPCG was further evaluated using representative O/W emulsions. As shown in Figure 8a,b, the milky emulsion passed through CPCG under gravity, and a clear filtrate was collected, visually confirming the effective removal of dispersed oil droplets. Optical microscopy was further used to examine the emulsions before and after filtration. Before separation, abundant micrometer-sized oil droplets were observed in both the low-viscosity cyclohexane/water emulsion and the high-viscosity castor oil/water emulsion (Figure 8c,d). After filtration through CPCG, almost no obvious oil droplets remained in the corresponding filtrates (Figure 8e,f). The droplet size distribution was also markedly reduced after separation (Figure 9a,b), further demonstrating the efficient interception of emulsified oil droplets by CPCG.

**Figure 10:**
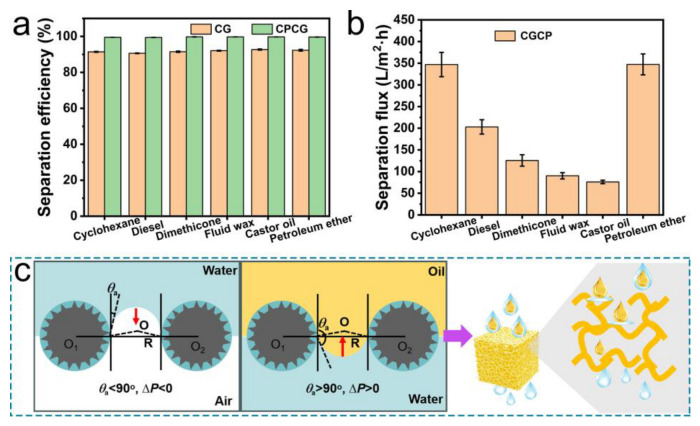
O/W emulsion (**a**) separation efficiencies and (**b**) separation flux of the aerogel; (**c**) emulsion separation mechanism of the CPCG.

To highlight the effect of CS/PVA modification, the separation efficiencies of CG and CPCG toward O/W emulsions prepared from six different oils, including cyclohexane, diesel, dimethyl silicone oil, liquid paraffin, castor oil, and petroleum ether, were compared. As shown in Figure 10a, CG exhibited limited separation efficiencies of only 90–92% for these emulsions. In contrast, CPCG achieved high separation efficiencies of 99–100% for all tested emulsions, confirming that the CS/PVA coating plays a crucial role in enhancing emulsion separation performance. Meanwhile, CPCG showed different flux values depending on the oil phase. The cyclohexane/water and petroleum ether/water emulsions exhibited the highest flux, reaching approximately 350 L/m^2^·h, whereas the castor oil/water emulsion showed the lowest flux of about 75 L/m^2^·h (Figure 10b). This difference is mainly associated with the viscosity of the oil phase and the droplet characteristics of the corresponding emulsions. Higher oil viscosity generally increases flow resistance and may enhance pore-blocking effects, thereby reducing the permeation flux.

The separation mechanism of CPCG can be attributed to the synergistic effect of its hydrophilic surface chemistry and interconnected porous structure (Figure 10c). Before emulsion filtration, CPCG can be rapidly wetted by water, allowing the aqueous phase to penetrate and occupy the porous channels. After water pre-wetting, a stable hydration layer forms on the pore walls, endowing the aerogel with underwater oil-repellent properties [29,30]. During filtration, water can continuously permeate through the interconnected channels, whereas emulsified oil droplets are intercepted by the hydrated oil-repellent interface and further hindered by the tortuous porous framework. As a result, CPCG enables efficient gravity-driven separation of the continuous water phase from dispersed oil droplets in O/W emulsions.

**Figure 11:**
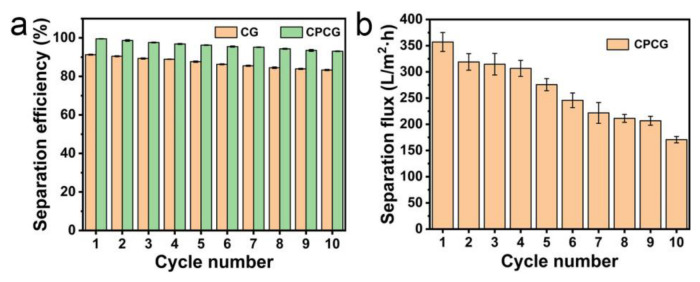
(**a**) Separation efficiencies and (**b**) separation flux of the aerogel during ten consecutive cyclohexane/water emulsion separation cycles.

To evaluate the cyclic stability of CPCG, cyclohexane/water emulsion was selected as a representative system for ten consecutive gravity-driven separation cycles. As shown in Figure 11a, the separation performance of CG gradually deteriorated with increasing cycle number, and its separation efficiency decreased from 91% to 86%. This decline may be attributed to oil adsorption and accumulation on the pore walls, partial blockage of water transport channels, and structural fatigue of the aerogel framework during repeated filtration. In contrast, CPCG exhibited much better cyclic stability, maintaining a separation efficiency above 93% after ten cycles, although its initial efficiency was higher than 99%. Meanwhile, the flux of CPCG decreased gradually during cycling and remained approximately 170 L/m^2^·h after the tenth cycle (Figure 11b). The slight decline in flux is likely related to the gradual accumulation of residual oil droplets and increased flow resistance within the porous channels.

Overall, CPCG shows a favorable balance among separation efficiency, flux, recyclability, and structural robustness. Compared with reported aerogel membranes, porous foams, and composite filtration materials summarized in Table 1, CPCG may not exhibit the highest flux, but it combines high separation efficiency, gravity-driven operation, facile preparation, good wet-state elasticity, and stable underwater oil repellency. These features make CPCG a promising low-energy and reusable material for O/W emulsion separation.

## 3. Conclusions

In this work, a hydrophilic and underwater oleophobic CPCG was successfully prepared through a facile dip-coating, GA crosslinking, and NaOH-assisted solidification strategy. A cross-linked CS/PVA coating was formed on the cellulose framework, thereby improving structural integrity, hydrophilicity, underwater oleophobicity, and wet-state resilience. CPCG exhibited a high underwater oil contact angle of 153.8°, which remained above 153° after 30 min, and retained 99% of its original height after 30 compression–recovery cycles. Benefiting from the stable hydration layer, interconnected porous structure, and reinforced framework, CPCG efficiently separated light and heavy oil/water mixtures as well as various O/W emulsions. The separation efficiencies for different emulsions reached 99–100%, and the efficiency remained above 93% after ten consecutive cyclohexane/water emulsion separation cycles. The enhanced performance originates from the synergistic effect of the cellulose porous skeleton and the CS/PVA hydrophilic coating: the cellulose framework provides continuous water transport channels, while the coating forms a hydrated barrier that suppresses oil adhesion and penetration underwater. This study offers a green, low-cost, and scalable approach for constructing biomass-based aerogels for oily wastewater treatment and emulsion separation.

## 4. Materials and Methods

### 4.1. Materials

Cellulose, CS, sodium hydroxide (NaOH), urea, N,N′-methylene bisacrylamide (MBA), and GA were purchased from Tianjin Zhiyuan Chemical Reagent Co., Ltd. (Tianjin, China). PVA (1788) was purchased from Shanghai Aladdin Chemical Co., Ltd. (Shanghai, China). Acetic acid was supplied by Tianjin Bodi Chemical Co., Ltd. (Tianjin, China). Chloroform and dimethicone were purchased from Chengdu Kelong Chemical Co., Ltd. (Chengdu, China). Cyclohexane, petroleum ether, and liquid wax were purchased from Tianjin Baishi Chemical Co., Ltd. (Tianjin, China). Diesel fuel was obtained from Sinopec Co., Ltd. (Beijing, China). Castor oil was purchased from Tianjin Oubokai Chemical Co., Ltd. (Tianjin, China).

### 4.2. Preparation of CG

CG was prepared through low-temperature dissolution, network reinforcement, and freeze-drying. Briefly, 5 g cellulose powder was dispersed in a pre-cooled NaOH/urea aqueous solution with a mass ratio of NaOH/urea/H_2_O = 7:13:80 under continuous stirring, and the suspension was frozen at low temperature for 12 h to obtain a homogeneous cellulose solution (5 wt%). Subsequently, 25 g of the cellulose solution was mixed with 0.4 g of MBA, which served as a physical network-reinforcing additive rather than a chemically polymerized crosslinker. The amide groups of MBA could form hydrogen-bonding interactions with cellulose hydroxyl groups, thereby helping stabilize the regenerated cellulose network. The mixture was stirred at room temperature for 3 h, poured into a mold, and allowed to stand for another 3 h. After demolding, the obtained cellulose hydrogel was immersed in distilled water to remove residual NaOH and urea until the pH of the washing water reached approximately 7. Finally, the washed hydrogel was frozen at −20 °C for 12 h and freeze-dried at −50 °C for 48 h to obtain CG.

### 4.3. Preparation of CPCG

**Figure 12:**
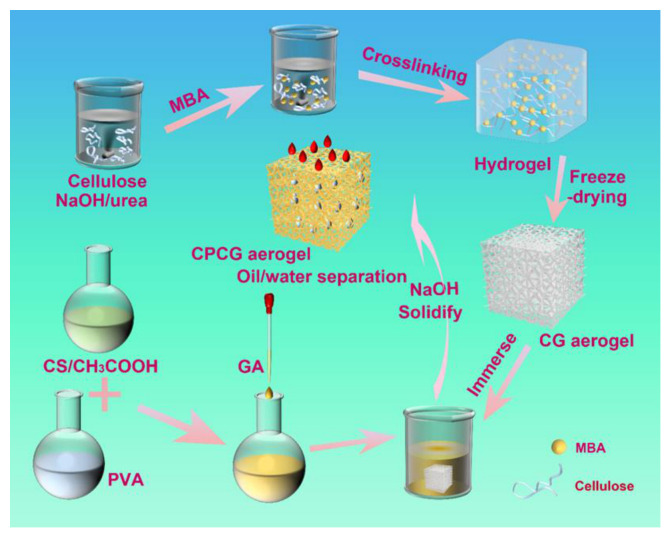
Schematic illustration of the preparation process of CPCG.

CPCG was fabricated by impregnating the CG with a CS/PVA mixed coating solution, followed by GA crosslinking and NaOH solidification (Figure 12). First, 0.5 g of chitosan was dissolved in 99.5 g of 0.5 vol% acetic acid aqueous solution and stirred at 500 rpm in a 50 °C water bath for 30 min to obtain a 0.5 wt% CS solution. Meanwhile, 0.5 g of PVA was dissolved in 99.5 g of deionized water in a 95 °C water bath for 2 h with occasional stirring to prepare a 0.5 wt% PVA solution. The CS solution and PVA solutions were then mixed at a mass ratio of 1:1 under magnetic stirring. The commercial GA solution was diluted to 1 wt% before use, and 40 μL of the diluted GA solution was added to every 1.0 g of CS/PVA coating solution to initiate crosslinking. During this process, the aldehyde groups of GA mainly reacted with the amino groups of CS to form Schiff base linkages, while possible interactions with the hydroxyl groups of PVA or cellulose also helped stabilize the coating network. The as-prepared CG was immersed in the CS/PVA/GA coating solution for 30 min to allow sufficient penetration into the internal porous network. After immersion, the coated aerogel was removed, reacted at room temperature for 3 h, immersed in 2 wt% NaOH solution for 1 h to solidify and stabilize the coating layer, and finally dried at room temperature to obtain CPCG. The CS/PVA coating gel prepared without cellulose aerogel was denoted as CPG.

### 4.4. Characterization

The surface morphology of the aerogels was observed using scanning electron microscopy (SEM, MERLIN Compact, Zeiss, (Jena, Germany)) at an accelerating voltage of 20 kV. Fourier transform infrared spectroscopy (FT-IR, VERTEX 70, Bruker, (Ettlingen, Germany)) was recorded using KBr pellets in the range of 4000–400 cm^−1^. Thermogravimetric analysis (TG, SDTQ600, TA Instruments (New Castle, DE, USA)) was performed from 25 to 800 °C at a heating rate of 10 °C/min under a nitrogen atmosphere. Wettability was evaluated using a contact angle analyzer (JC2000D4, Zhongchen, (Shanghai, China)). In the contact angle measurements, the volumes of the water and oil droplets were both 2 μL. All contact angles were averaged from measurements on multiple samples and at multiple positions. The droplet size distributions of the emulsions before and after separation were measured using a Zeta potential analyzer (Zetasizer Nano ZS90, Malvern, (Malvern, UK)). The compressive stress–strain behavior of wet aerogels was tested using a universal testing machine (FL4204GDL, Sturdy, Shanghai, China) at a compression speed of 5 mm/min. Cylindrical samples with a diameter of 1.5 cm and a height of 1 cm were used for the compression tests. The fully wetted aerogels were compressed and released repeatedly for 30 cycles, and the height retention ratio was calculated by comparing the recovered height after cycling with the initial height. The oil content in the O/W emulsions and filtrates was measured using an infrared spectrophotometric oil meter (JC-OIL-6, Juchuang Environmental Protection, Qingdao, China).

### 4.5. Oil/Water and Emulsion Separation Tests

Preparation of O/W emulsions: The oil and water phases were mixed at a volume ratio of 1:10, and 1 wt.% Tween 60 was added as an emulsifier. The mixture was magnetically stirred for 3 h to obtain a stable milky-white emulsion. The selected oil phases included cyclohexane, diesel, dimethyl silicone oil, liquid paraffin, castor oil, and petroleum ether.

The emulsion separation experiment was performed using a self-made oil/water separation device. The device consisted of an upper feed tube, a fixture, an aerogel sample, a sealing gasket, and a lower receiving bottle. Photographs of the device are shown in Figure 9a,b. During each test, a cylindrical aerogel sample with a diameter of 1.5 cm and a height of 1 cm was fixed in the device, and 20 mL of emulsion was added for each run. The filtration process was driven solely by gravity without any external pressure, and the time required for complete filtration was recorded. The separation efficiency (%) and separation flux (L/m^2^·h) were calculated according to Equations (1) and (2) [38], respectively:

(1) $$\mathrm{Separation}\;\mathrm{efficiency}=\left(\frac{C_{0}-C_{f}}{C_{0}}\right)\times 100\%$$

(2) $$\mathrm{Separation}\;\mathrm{flux}=\frac{V}{A\times t}$$

where *C*_0_ and *C*_f_ represent the oil concentrations in the feed emulsion and filtrate, respectively. *V* (L) is the volume of the emulsion, *A* (m^2^) is the effective filtration area, and *t* (h) is the separation time.

The oil absorption-release performance of the aerogel was evaluated in a transparent beaker. Specifically, oil was dropped onto the water surface to form an oil layer, and the aerogel was then allowed to contact and absorb the oil layer. Subsequently, the oil-loaded aerogel was transferred into the aqueous phase and gently squeezed to observe the oil release process and the shape recovery behavior of the aerogel.

The cyclic stability of the aerogels was evaluated by repeating the emulsion separation process. Ten consecutive cyclohexane/water emulsion separation cycles were conducted using the same aerogel sample. In each cycle, 20 mL of emulsion was added and filtered under identical gravity-driven conditions using the same separation device. After each separation, the filtrate was collected for oil concentration and droplet size measurements, and the separation time was recorded for flux calculation. The aerogel was then removed, gently squeezed, and reused for the next cycle.

## Figures and Tables

**Table 1:** Comparison of the O/W emulsion separation performance of materials.

Materials	Separation Efficiency	Flux	Cycles Retention	Refs
Chitosan-based aerogel membrane	>99%	>600 L/m^2^·h·bar	-	[31]
Aramid nanofiber aerogel membrane	98.1%	1940 L/m^2^·h·bar	10 cycles > 98%	[32]
Superhydrophobic melamine foam	99.25%	-	-	[33]
Facile construction of multifunctional bio-aerogel	69.76~99.25%	170~1921 L/m^2^·h	5 cycles > 80%	[34]
Zeolitic imidazolate framework-67 modified membranes	>99.6%	>311 L/m^2^·h	6 cycles > 99%	[35]
Sponge based on sodium alginate-loofah composite gels	>99.3%	>35 L/m^2^·h	5 cycles > 70%	[36]
Wood filter	96.32%	410 L/m^2^·h·bar	6 cycles > 90%	[37]
CPCG	>99.5%	76~350 L/m^2^·h	10 cycles > 93%	This work

## Data Availability

The original contributions presented in this study are included in the article. Further inquiries can be directed to the corresponding author.
